# The longitudinal relationships among adverse childhood experiences, lifestyle, and late-life depression: a latent growth mediation model

**DOI:** 10.3389/fpsyt.2025.1581908

**Published:** 2025-07-29

**Authors:** Xin Cai, Haojie Yin

**Affiliations:** ^1^ Clinical Medical College & Affiliated Hospital of Chengdu University, Chengdu University, Chengdu, China; ^2^ Department of Respiratory and Critical Care Medicine, Neijiang First People’s Hospital, Neijiang, China

**Keywords:** adverse childhood experiences, depression, lifestyle, middle-aged and older adults, mediating effects

## Abstract

**Background:**

Many studies have shown that adverse childhood experiences (ACEs) are one of the causes of depressive symptoms in middle-aged and elderly individuals, but the combined impact of ACEs and lifestyle factors on depression has not been fully explored.

**Methods:**

This study used data from the China Health and Retirement Longitudinal Study (CHARLS) from 2013, 2014, 2015, 2018, and 2020, including 10,835 middle-aged and elderly individuals. A latent variable growth curve model was used to analyze the trends of late-life depression and lifestyle changes. The mediating role of lifestyle in the relationship between ACEs and depression was also assessed.

**Results:**

ACEs were significantly associated with lower initial lifestyle levels (β = -0.138, *P* <.001) and higher depression severity (β = 0.253, *P* <.001). The mediation analysis results showed that the relationship between ACEs and the depression intercept was partially mediated by the initial level of lifestyle (β = 0.021, *P* <.001), and the relationship between ACEs and the depression slope was entirely mediated by the initial level of lifestyle (β = -0.010, *P* <.01). Different dimensions of ACEs had varying effects on depression.

**Conclusions:**

ACEs are associated with the severity of depression, partly due to lower lifestyle levels. Interventions focused on reducing ACEs and improving lifestyle could effectively reduce the incidence of depression in middle-aged and elderly populations.

## 1 Introduction

With the declining birth rate and increasing life expectancy, global population aging has emerged as a critical public health challenge (1). This trend is particularly pronounced in China, where projections indicate that by 2050, individuals aged ≥ge and ≥nd will reach 400 million and 150 million respectively (2). Such rapid demographic shift necessitates urgent attention to modifiable factors influencing late-life health outcomes (3). As a cornerstone of healthy aging strategies, mental health – particularly late-life depression – demands focused research (4). Depression remains the second leading global cause of disability (5, 6), with elderly populations facing unique vulnerabilities: while incidence rates are lower than younger groups (7), associated suicide rates and health burdens are disproportionately higher (8). Notably, depression in older adults exacerbates social disengagement, functional impairment, and healthcare costs (9–11), making it a priority concern for China’s aging society.

It is widely believed in academia that depression results from the combined influence of biological, psychological, and social factors, with early trauma being the most common and significant trigger (12, 13). In terms of early trauma, childhood adversity is a common pathway leading to long-term social, emotional, and cognitive dysfunctions, including depression (14, 15). Adverse childhood experiences (ACEs), also known as childhood adversity, refer to a series of negative events that occur during childhood, including abuse, neglect, substance abuse, and family dysfunction (16). In addition to the short-term effects on childhood and early adulthood health, some studies suggest that the impact of ACEs can persist into late adulthood and is associated with an increased risk of depression in the elderly population (17, 18). For example, adults with a history of parental substance abuse have a 69% higher risk of depression compared to their peers (19). Young women who have experienced childhood adversity, such as domestic violence, parental mental illness, and alcohol abuse, have a higher risk of depression than those who have not experienced such adversities (20). However, the mechanisms underlying this relationship remain not fully understood.

The life course model emphasizes that ACEs have direct or indirect effects on mental health in adulthood (21, 22). The direct effects arise from the adverse experiences themselves, while the indirect effects suggest that the relationship between ACEs and mental health (such as depression) may be mediated by certain factors, such as lifestyle, cognitive function, and life satisfaction (5, 18). A substantial body of research has shown a significant relationship between ACEs and health behaviors or lifestyles throughout life (23, 24). Lifestyle factors (such as diet, exercise, sleep, and harmful health behaviors) are important determinants of depression (25). This may be related to the fact that physiological stress responses exacerbate impulsivity and promote unhealthy emotional regulation mechanisms (26, 27). For example, ACEs are strongly associated with an increase in risky behaviors, such as eating disorders. Among adult samples seeking surgical treatment for obesity, one-fifth of individuals experienced sexual or physical abuse during childhood, and more than one-third experienced violence (28). However, diet is a key factor in the increased or decreased incidence of depression (29, 30). Anda et al. reported that ACEs are an important factor in the initiation of smoking during adolescence and the continuation of smoking into adulthood (31). A study in Europe also found that physical abuse, emotional neglect, and poor parental relationships have a positive impact on the likelihood of smoking and alcohol abuse in adulthood (24). Nurius et al. suggested that buffering factors for childhood adversity may include lifestyle behaviors, such as physical activity, which can reduce stress levels, improve physiological function, and thereby decrease the risk of depression, leading to better physical and mental health outcomes (26, 32). In summary, lifestyle factors may buffer or exacerbate the impact of ACEs on depression through various pathways.

The distinction between mediating and moderating effects is critical to our theoretical framework. A moderator influences the strength or direction of the relationship between an independent variable (e.g., ACEs) and a dependent variable (e.g., depression), whereas a mediator explains the pathway through which the independent variable affects the dependent variable (33, 34). In this study, we hypothesize lifestyle as a mediator rather than a moderator for two reasons: 1. Pathway-driven perspective: Existing evidence suggests that ACEs may shape health-related behaviors (e.g., smoking, poor diet) as maladaptive coping strategies (24), which in turn contribute to physiological dysregulation (e.g., inflammation, HPA axis dysfunction) and increased depression risk (35, 36). This aligns with a mediation model where lifestyle serves as a mechanism linking early trauma to later mental health outcomes. 2. Temporal precedence: Longitudinal data support the temporal sequence required for mediation: ACEs precede the development of lifestyle patterns, which subsequently influence depression trajectories in later life. In contrast, moderation would imply that lifestyle factors (e.g., exercise) alter the effect of ACEs on depression (e.g., buffering ACEs-related risk only among physically active individuals), a hypothesis less supported by prior life-course studies.

Previous studies have established a link between ACEs and depression, and lifestyle factors may play a key role in this relationship. However, our understanding of the underlying mechanisms remains limited (37), especially in the context of depression trajectories over time. Depression is not a static condition, its severity and progression vary among individuals (38). In addition to public efforts to eliminate the effects of ACEs themselves, it is crucial to understand how childhood adversity influences the initial levels of depression and its developmental trajectory, as well as the role that lifestyle factors may play in this relationship. This understanding is vital for identifying modifiable factors that can be targeted for intervention later in life to mitigate the impact of ACEs across the life course. Therefore, to fill the gap in the literature, this study aims to explore the relationship between ACEs and the development of depression in middle-aged and older adults in China, and to investigate the potential longitudinal mediating role of lifestyle factors in this relationship. The study proposes the following hypotheses: (i) compared to middle-aged and older adults without childhood adversity, those who have experienced adversity will have more severe initial levels of depressive symptoms and a faster progression of depression in late life; (ii) Lifestyle factors play a longitudinal mediating role in the relationship between ACEs and depression; (iii) There are differences in the relationship between different dimensions of childhood adversity and depression.

## 2 Materials and methods

### 2.1 Data source

The data for this study comes from the China Health and Retirement Longitudinal Study (CHARLS), which is a study targeting adults aged 45 and above in China. The baseline CHARLS data were collected in 2011, followed by four subsequent follow-up surveys in 2013, 2015, 2018, and 2020. The CHARLS project uses a multi-stage stratified probability sampling method with proportional size, covering 28 provinces in mainland China, 450 villages/community committees, and approximately 10,000 households. It gathers extensive information, including data on residents’ demographics, social characteristics, health status, economic status, family structure, and social activities. The life history module collected in 2014 further includes information on the respondents’ childhood family history, health history, education history, wealth history, and employment history (39). Therefore, CHARLS reflects the overall situation of the middle-aged and elderly population in China and has good national representativeness. The CHARLS project was approved by the Biomedical Ethics Committee of Peking University (IRB00001052-11015), and all participants provided written informed consent. This study was authorized by the CHARLS database.

**Figure 1:**
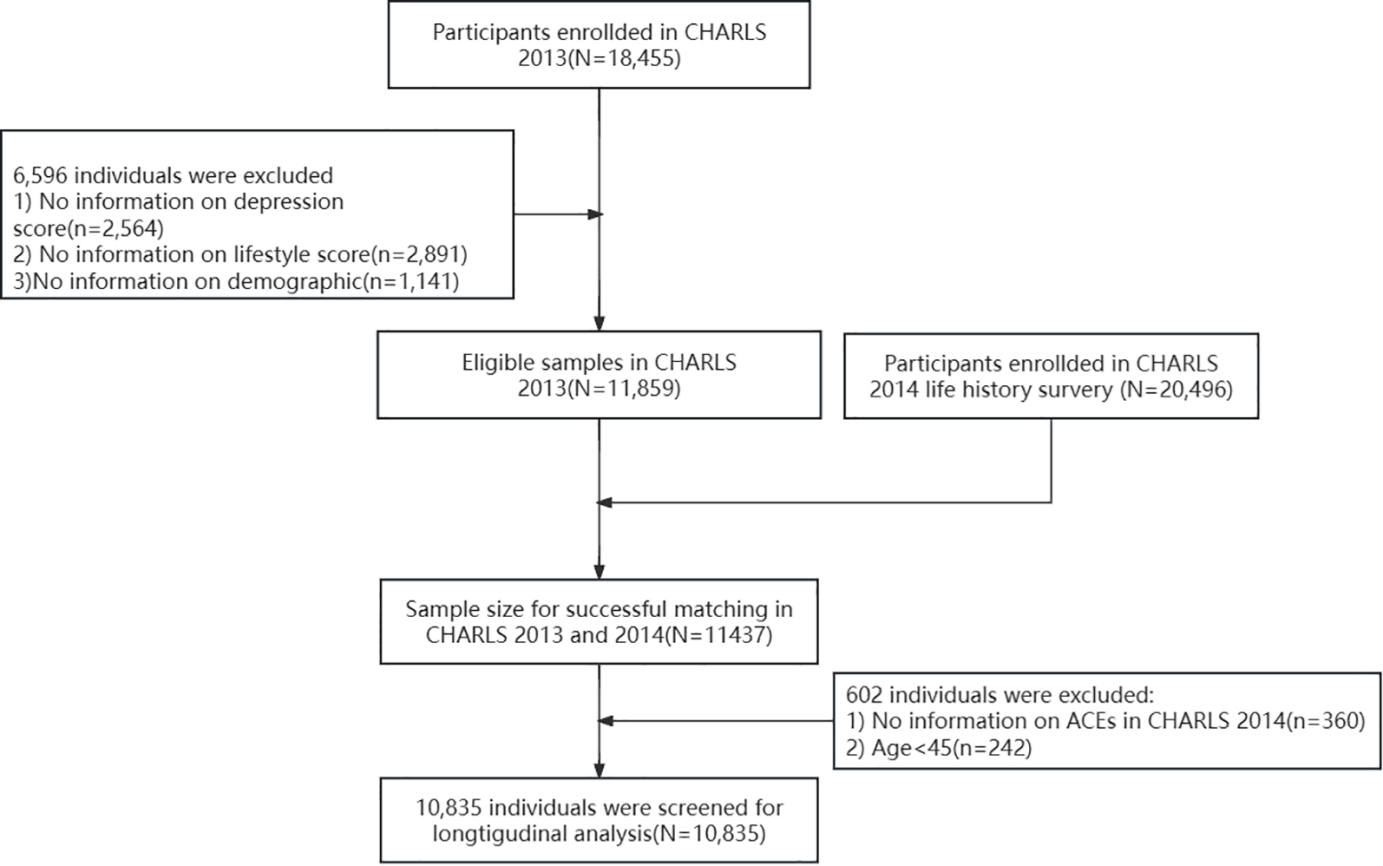
Flow chart of the selected participants from the CHARLS.

The five waves of CHARLS data (2013 (Wave 1), 2014(Wave 2), 2015 (Wave 3), 2018 (Wave 4), and 2020 (Wave 5)) were included in this study. Comprehensive information on lifestyle and depression were collected at wave 1, 3, 4, and 5. Demographic characteristics of the participants were collected in the first wave, while ACE information was gathered in the life history module of the second wave. There were a total of 11,859 eligible participants in 2013 (excluding those with missing data on lifestyle, depression, and demographic information (n=6,596)). The exclusion of participants with missing data in baseline variables (2013) was based on two considerations: 1. Missingness in key variables (e.g., lifestyle, depression) at baseline may reflect non-random patterns (e.g., refusal to report sensitive information), and complete-case analysis reduces potential bias from unmeasured confounding. 2. The large proportion of missing data could lead to unstable imputation results if traditional methods (e.g., mean/mode imputation) were applied, whereas advanced multiple imputation would require stronger assumptions about missing data mechanisms. Subsequently, we matched the 2013 data with the life history module data collected in 2014 based on ID, excluding 242 participants aged under 45 and 782 participants with incomplete ACE information. Finally, this study selected a cohort of 10,835 CHARLS participants. To minimize cohort effects, participants with missing data in the subsequent three waves (2015, 2018, and 2020) were included in the analysis. However, data from participants who joined the CHARLS study after 2013 were not included in the analysis. A detailed flow chart of the sample selection process is shown in **Figure 1**.

### 2.2 Measures

#### 2.2.1 Depression

Depression was measured using the revised version of the Center for Epidemiologic Studies Depression Scale-10 (CESD-10), which is an effective screening tool for depressive symptoms in the middle-aged and elderly population in China (40). The CESD-10 consists of 10 items, each measuring the frequency of a specific type of negative emotion. Responses range from “Rarely or none of the time (<1 day)” to “Most or all of the time (5–7 days),” with scores assigned from 1 to 4. The two positively worded items in the scale (“I feel hopeful about the future” and “I feel happy”) are reverse-scored. The total score ranges from 10 to 40, with higher scores indicating more severe depressive symptoms. In the sample of this study, the CESD-10 demonstrated high internal consistency (Cronbach’s alpha = 0.796) and validity (KMO = 0.880, *P* <.01).

#### 2.2.2 Lifestyle

We constructed the lifestyle score using five factors: smoking, alcohol consumption, social participation, physical activity, and sleep duration. Each factor was categorized as “Yes” (score = 1) or “No” (score = 0). If the participant had never smoked, the smoking status score was 1, otherwise, it was 0. If the participant had not consumed alcohol in the past year, the alcohol consumption status score was 1, otherwise, it was 0. If the participant had engaged in social activities in the past month (such as socializing with friends, playing mahjong/chess/cards, participating in community clubs, engaging in physical activities, joining other types of clubs, taking part in community-related organizations, volunteering or doing charity work, or attending educational or training courses), each activity was assigned 1 point, otherwise, it was 0. If the participant slept for more than 6 hours at night, the sleep duration score was 1, otherwise, it was 0. If the participant engaged in physical activity for more than 10 minutes per week, the physical activity score was 1, otherwise, it was 0. We combined the scores of these five factors into an overall lifestyle score, ranging from 0 to 15. A higher score indicates a healthier lifestyle (41).

#### 2.2.3 Adverse childhood experiences

The life history module of the 2014 CHARLS study, based on the World Health Organization’s Adverse Childhood Experiences International Questionnaire (ACE-IQ), collected data on the adverse childhood experiences that participants had encountered before the age of 18. We extracted 13 ACEs from the 2014 dataset, with each ACE defined as a binary variable (0 = no, 1 = yes or 0 = good, 1 = bad). Based on the cumulative scoring framework, the responses to each question were summarized to generate the participants’ cumulative ACE score, with a total score ranging from 0 (no ACE exposure) to 13 (exposed to all ACEs) (42). In addition, we used exploratory factor analysis to categorize the 13 ACEs into three distinct dimensions: conventional ACEs (physical abuse, emotional neglect, domestic violence, family crime, family mental illness, family substance abuse, parental divorce/separation), expanded ACEs (unsafe living environment, peer bullying, and poor childhood friendships), and new ACEs (early parental death, early sibling death, and parental disability) (43). Similarly, the responses to each item were scored using the cumulative scoring method to generate the cumulative ACEs score for each dimension. The detailed definitions of the ACEs indicators can be found in **Supplementary material Table 1**.

#### 2.2.4 Covariate information

Demographic characteristics of the 10,835 respondents who participated in the study from 2013 to 2020 were analyzed. The demographic variables measured at baseline (2013) included age, gender (0 = female, 1 = male), marital status (0 = unmarried/divorced/separated/widowed, 1 = married/cohabiting), ethnicity (0 = other, 1 = Han), official household registration status in China (0 = non-agricultural, 1 = agricultural), educational level (0 = illiterate, 1 = primary school or below, 2 = middle school/technical secondary school, 3 = high school or above), and birthplace (0 = urban, 1 = rural). These covariate variables were included in our research model.

#### 2.2.5 Statistical analysis

Descriptive statistical analysis of the characteristics of the study sample was conducted using IBM SPSS version 25.0. Continuous variables were represented by mean and standard deviation, while categorical variables were represented by frequency and percentage. Latent Growth Curve Modeling (LGCM) was used to capture the trajectories of changes in respondents’ depression and lifestyle scores, where the intercept factor (I) represented the initial level of both indicators at baseline (2013), and the slope factor (S) represented the rate of change in both indicators over the four observation periods. Finally, we used LGCM to analyze the mediation effect, which provided the mean estimates of the intercept and slope factors as fixed effects. We addressed this research question in two steps. First, we examined the direct relationship between the ACE total score/dimensions and the development of depression while controlling for a set of covariates (e.g., age, gender, education level, etc.) in Model 1. Then, we added lifestyle factors to Model 1 to test the mediation hypothesis of the study (Model 2). The goodness of fit of the model was assessed using the following indicators: Tucker-Lewis index(TLI), comparative fit index (CFI) > 0.9, root mean square error of approximation (RMSEA) < 0.07, standardized root mean square residual (SRMR) < 0.05, and chi-square test (χ^2^). Due to the large sample size in the study, the χ^2^ result was not ideal. All mediation analyses were conducted using the Mplus 8.0 statistical software. Missing values were handled using Full Information Maximum Likelihood (FIML) estimation, which allows for model parameters to be estimated based on all available data. Robust maximum likelihood estimator was provided when the data are non-normality and missing at random. The indirect effects were estimated based on 10,000 bootstrap samples of the maximum likelihood estimation.

## 3 Results

### 3.1 Descriptive statistics

The statistical characteristics of the study sample are presented in **Table 1**. The average age of the respondents was 59.25 years, 53.68% of the respondents were female, 89.53% were married. Notably, the sample exhibited distinct sociodemographic patterns: 91.83% of participants were born in rural villages, and 79.31% held agricultural household registration (hukou). Education levels were relatively low, with 23.22% being illiterate, 44.37% attaining primary school or below, 23.17% completing junior high school/technical secondary school, and only 9.24% achieving senior high school or higher education. This educational distribution suggests potential socioeconomic vulnerability that may contextualize subsequent lifestyle behaviors and mental health outcomes. The average CESD-10 scores in 2013, 2015, 2018, and 2020 were 17.86, 18.10, 18.93, and 19.29, respectively. The average lifestyle scores were 3.22, 3.33, 3.65, and 3.87, indicating that the respondents’ lifestyle gradually improved over time, while depressive symptoms progressively worsened. In addition, the average ACE score was 1.93 (SD = 1.53), with 28.88% of respondents having experienced physical abuse, 30.13% having had poor childhood friendships, and 26.28% having experienced emotional neglect within the family. More details about ACEs of respondents are shown in **Table 1** and **Supplementary material Figure 1**.

**Table 1:** Descriptive characteristic of participants.

Variables	Levels	Observed N (%)	Mean (SD)	Minimum	Maximum	Missing N (%)
ACEs	scores		1.93 (1.53)	0	10	
Conventional ACEs (0-7)	scores		0.84 (0.91)	0	5	
Physical abuse	yes	3,129 (28.88)				
Emotional neglect	yes	2,847 (26.28)				
Domestic violence	yes	941 (8.68)				
Family crime	yes	39 (0.36)				
Mental illness in the family	yes	1,371 (12.65)				
Household Substance Abuse	yes	750 (6.92)				
Parents divorced or separated	yes	80 (0.74)				
Expanded ACEs (0-3)	scores		0.54 (0.70)	0	3	
Unsafe living conditions	yes	881 (8.13)				
Peer bullying	yes	1,751 (16.16)				
Bad friendship experiences	yes	3,265 (30.13)				
New ACEs (0-3)	scores		0.54 (0.70)	0	3	
Parental death before age 17	yes	1,832 (16.91)				
Death of a sibling before age 17	yes	1,795 (16.57)				
Parental disability	yes	2,297 (21.20)				
Depression (10-40)						
	w2013		17.86 (5.77)	10	40	
	w2015		18.10 (6.46)	10	40	631 (5.82)
	w2018		18.93 (6.65)	10	40	566 (5.22)
	w2020		19.29 (6.60)	10	40	1448 (13.36)
Lifestyle (0-15)						
	w2013		3.22 (1.48)	0	13	
	w2015		3.33 (1.53)	0	12	631 (5.82)
	w2018		3.65 (1.47)	0	12	566 (5.22)
	w2020		3.87 (1.49)	0	11	1448 (13.36)
Demographic characteristics						
Age (45-103)			59.25 (8.76)	45	103	
Gender						
Male		5,019 (46.32)				
Female		5,816 (53.68)				
Married		9,701 (89.53)				
Education						
Illiterate		2,516 (23.22)				
Primary school and below		4,807 (44.37)				
Junior high school		2,511 (23.17)				
Senior high school and above		1,001 (9.24)				
Han nationality		10,049 (92.75)				
Current agricultural hukou status		8,593 (79.31)				
Born in rural village		9,950 (91.83)				

Sample *N* =10,835. ACEs, Adverse Childhood Experiences.

### 3.2 The growth trajectories of depression and lifestyle

The unconditional LGCM model for depression scores and lifestyle scores demonstrated good fit: χ^2^/df =6.88, CFI=0.995, TLI=0.993, RMSEA=0.023, SRMR=0.017. The average intercept (baseline) of the depression scores was 17.837 (*P* <.001), and the average slope (change) was 0.192 (*P* <.001), indicating that with the passage of time, the depression score increased by 0.192 in each wave. The average estimated score for lifestyle (intercept) was 3.216 (*P* <.001), and the average score for the slope (change) was 0.081 (*P* <.001), indicating that with the passage of time, the lifestyle score increased by 0.081 in each wave. The above results indicate that depressive symptoms in middle-aged and elderly individuals in China tend to worsen with age, while lifestyle improves gradually with increasing age. More details about unconditional LGCM are presented in **Supplementary material Table 2**.

### 3.3 Direct association between ACEs and depression

**Figure 2:**
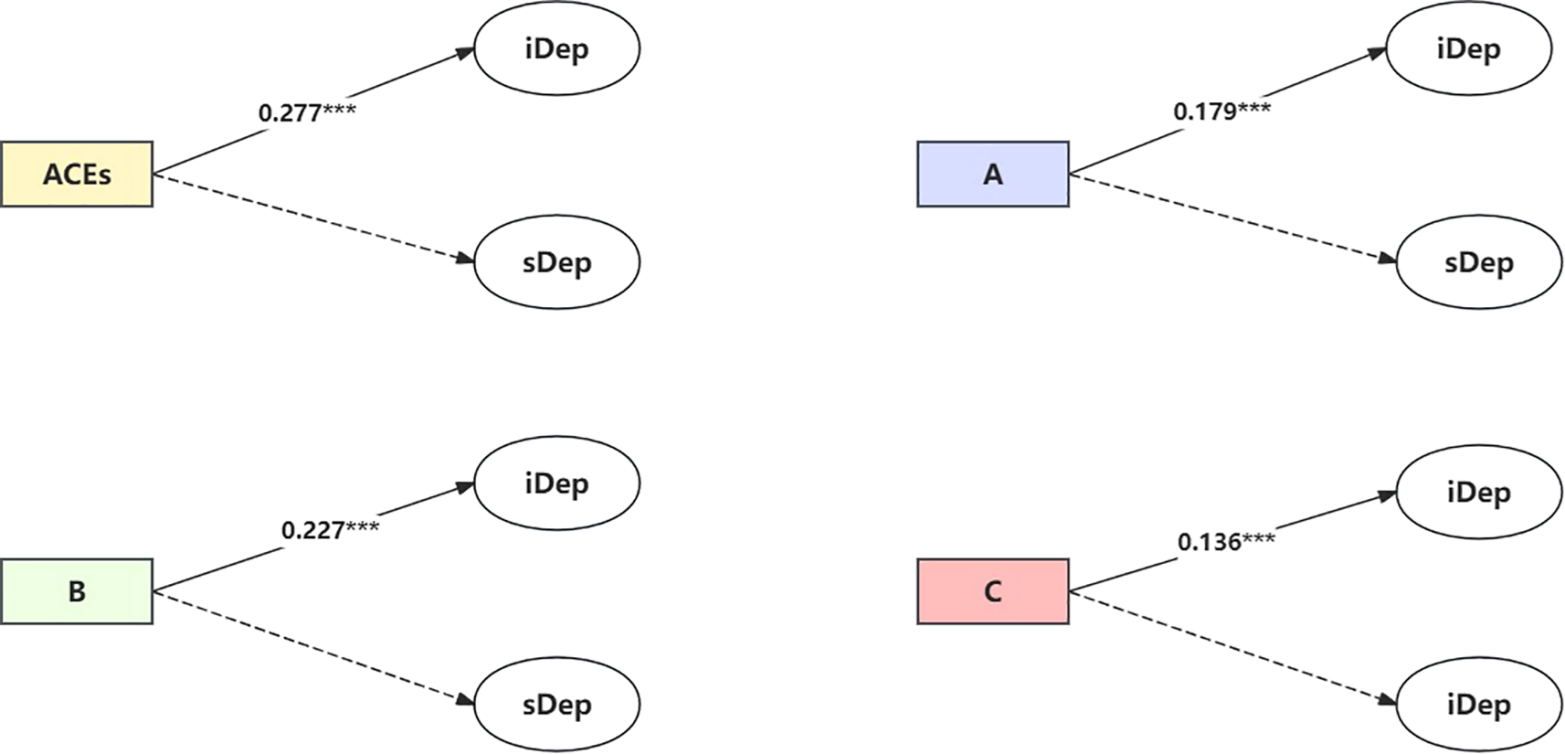
Direct association between ACEs/ACEs dimensions and depression. Note: solid arrow estimates in the model are statistically significant at the 95% significance level, and dotted arrows are not (see **Table 2** Model 1). iDep, Depression intercept; sDep, Depression slope. ACEs, Adverse Childhood Experience; **(A)** Conventional adverse childhood experiences; **(B)**, Expanded adverse childhood experiences; **(C)**, New adverse childhood experiences. Model: adjusted for demographic characteristic (age, gender, marital status, etc). ^***^ *P*<.001.

After adjusting for the demographic characteristics of the respondents (age, gender, marital status, education level, ethnicity, birthplace, etc.), the direct association between ACEs and depressive symptoms is presented in **Table 2** (Model 1) and **Figure 2**. The results indicated that, compared to individuals who had not experienced childhood adversity, those with more adverse experiences had higher baseline depression levels (β = 0.277, *P* <.001). However, ACEs were not associated with the slope (rate of change) of depression symptoms (β = -0.030, *P* = 0.112).

**Table 2:** Latent growth model on ACEs (total score and dimensions), lifestyle, and depression.

Variables	Model 1: Direct effect						Model 2: Indirect effect					
	Intercept			Slope			Intercept			Slope		
	β	SE	R^2^	β	SE	R^2^	β	SE	R^2^	β	SE	R^2^
ACEs	0.277^***^	0.011	0.136	-0.030	0.019	0.017	0.253^***^	0.013	0.152	-0.005	0.023	0.142
Lifestyle i							-0.150^***^	0.014		0.073^**^	0.024	
Lifestyle s							0.072^*^	0.034		-0.353^***^	0.062	
A	0.179^***^	0.011	0.092	-0.027	0.019	0.017	0.159^***^	0.013	0.114	-0.002	0.022	0.142
Lifestyle i							-0.166^***^	0.014		0.073^**^	0.024	
Lifestyle s							0.079^*^	0.035		-0.354^***^	0.062	
B	0.227^***^	0.011	0.111	0.005	0.019	0.016	0.207^***^	0.013	0.131	0.028	0.024	0.141
Lifestyle i							-0.158^***^	0.014		0.076^**^	0.002	
Lifestyle s							0.076^*^	0.024		-0.353^***^	0.062	
C	0.136^***^	0.011	0.079	-0.033	0.019	0.017	0.127^***^	0.012	0.105	-0.034	0.022	0.145
Lifestyle i							-0.174^***^	0.014		0.073^**^	0.024	
Lifestyle s							0.089^*^	0.036		-0.357^***^	0.063	
Fit indices	ACEs	A		B	C		ACEs	A		B	C	
*X* ^2^(*df*)	66.76(11)	65.35(11)		64.82(11)	69.54(11)		523.26(33)	558.81(33)		521.40(33)	559.09(33)	
RMSEA	0.022	0.021		0.021	0.022		0.037	0.038		0.037	0.038	
CFI	0.996	0.996		0.996	0.996		0.981	0.979		0.981	0.979	
TLI	0.994	0.994		0.994	0.993		0.975	0.973		0.975	0.972	
SRMR	0.021	0.020		0.020	0.021		0.039	0.042		0.039	0.042	

ACEs, Adverse Childhood Experiences. A, Conventional adverse childhood experiences; B, Expanded adverse childhood experiences; C, New adverse childhood experiences. CFI, comparative fit index. TLI, Tucker-Lewis index. RMSEA, root mean square error of approximation. SRMR, standardized root mean residual. ^***^ *P*<.001, ^**^ *P*<.01, ^*^ *P*<.05.

The results of the direct associations between ACEs dimensions and depression indicated that conventional ACEs (abuse, neglect, domestic violence), expanded ACEs (unsafe living environments, bullying, poor friendship experiences), and new ACEs (parental death, sibling death, and parental disability) were all positively correlated with the initial level (intercept) of depression (β = 0.179, 0.227, and 0.136, *P* <.001), but were not associated with the slope (rate of change) of depression symptoms.

### 3.4 Indirect association between ACEs and depression through lifestyle factors

**Figure 3:**
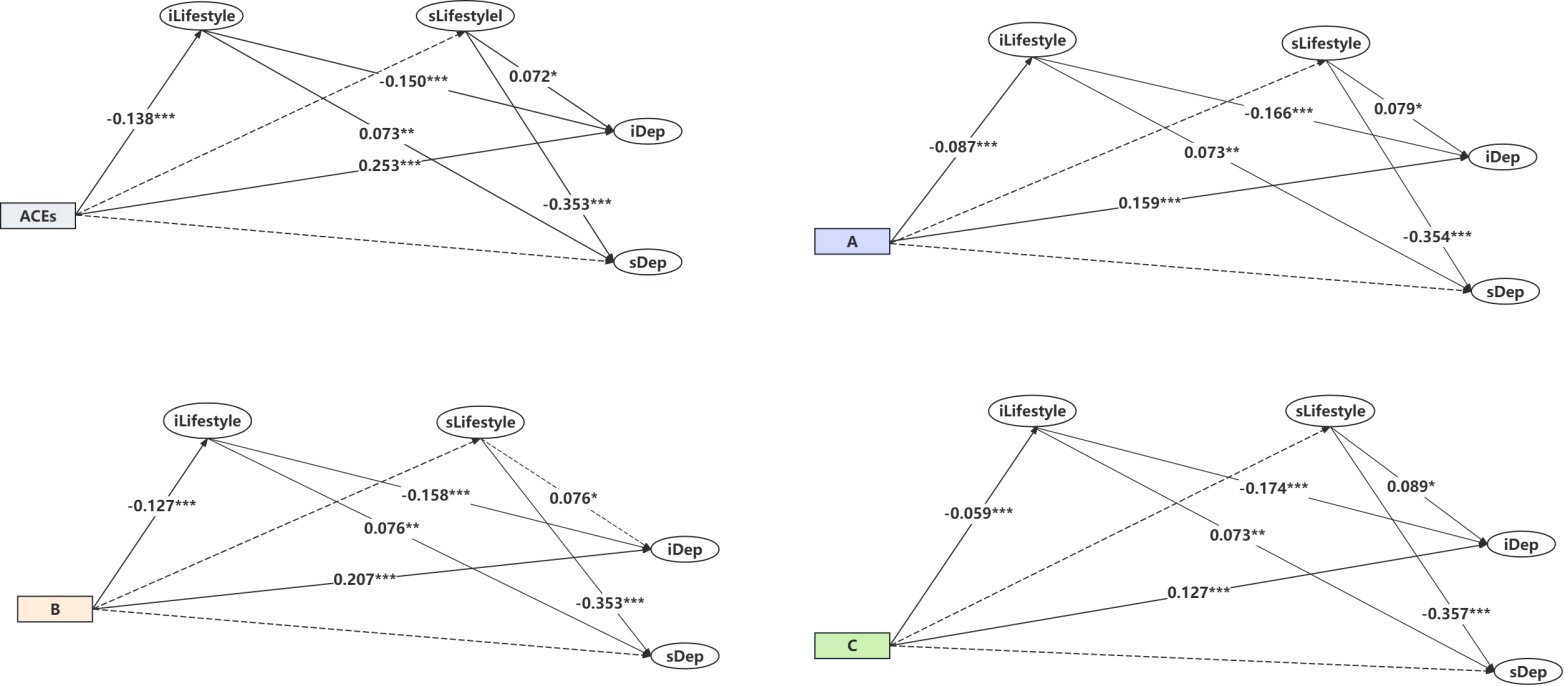
Indirect association between ACEs/ACEs dimensions and depression. Note: solid arrow estimates in the model are statistically significant at the 95% significance level, and dotted arrows are not (see **Table 2** Model 2). iDep, Depression intercept. sDep, Depression slope. ACEs, Adverse Childhood Experience. **(A)**, Conventional adverse childhood experiences. **(B)**, Expanded adverse childhood experiences. **(C)**, New adverse childhood experiences. Model: adjusted for demographic characteristic (age, gender, marital status, etc). ^***^ *P*<.001, ^**^ *P*<.01, ^*^ *P*<.05.

The indirect association between ACEs and depression is shown in **Table 2** (Model 2) and **Figure 3** after adjustment for demographic characteristics (age, gender, marital status, education level, ethnicity, birthplace, etc.) of participants. The results indicated that ACEs were positively correlated with the initial level of depression (β = 0.253, *P* <.001) and negatively correlated with the initial level of lifestyle (β = -0.138, *P* <.001). The initial level of lifestyle was negatively correlated with the depression intercept (β = -0.150, *P* <.001) and positively correlated with the depression slope (β = 0.073, *P* <.01). The slope of lifestyle was positively correlated with the depression intercept (β = 0.072, *P* <.05) and negatively correlated with the depression slope (β = -0.353, *P* <.001).

As for the dimensions of ACEs, conventional ACEs were positively correlated with the depression intercept (β = 0.159, *P* <.001) and negatively correlated with the lifestyle intercept (β = -0.087, *P* <.001). Expanded ACEs were positively correlated with the depression intercept (β = 0.207, *P* <.001) and negatively correlated with the lifestyle intercept (β = -0.127, *P* <.001). New ACEs were positively correlated with the depression intercept (β = 0.127, *P* <.001) and negatively correlated with the lifestyle intercept (β = -0.059, *P* <.001). More details can be found in **Table 2** (Model 2) and **Figure 3**. In summary, the findings suggest that the lifestyle intercept mediated the relationship between ACEs and depression (both intercept and slope).

The direct and indirect effects of ACEs on depression are shown in **Table 3** (results from **Table 2**, Model 2). The total association between ACEs and baseline depression severity was 0.277 (*P* <.001). The indirect effect of ACEs on baseline depression severity through the lifestyle intercept was 0.021 (*P* <.001), accounting for 7.5% of the total effect (= 0.021/0.277). The direct effect of ACEs on baseline depression severity was 0.253 (*P* <.001), accounting for 91.3% of the total effect (= 0.253/0.277). The indirect effect of ACEs on the depression slope through the baseline lifestyle was -0.010 (*P* <.01), and no direct effect was found between ACEs and the depression slope.

**Table 3:** Direct and indirect effects of ACEs (total score and dimensions) on depression in later life.

Effect	Intercept			Slope		
	β	SE	95% CI	β	SE	95% CI
Direct effect						
ACEs-Dep	0.253^***^	0.013	0.228 to 0.278	-0.005	0.023	-0.051 to 0.039
A-Dep	0.159^***^	0.013	0.134 to 0.184	-0.002	0.022	-0.046 to 0.040
B-Dep	0.207^***^	0.013	0.181 to 0.232	0.028	0.024	-0.018 to 0.074
C-Dep	0.127^***^	0.012	0.103 to 0.150	-0.034	0.022	-0.077 to 0.009
Indirect effect						
ACEs-iLifestyle-Dep	0.021^***^	0.003	0.016 to 0.026	-0.010^**^	0.003	-0.017 to -0.004
ACEs-sLifestyle-Dep	0.003	0.003	0.000 to 0.012	-0.017	0.012	-0.043 to 0.003
A-iLifestyle-Dep	0.014^***^	0.002	0.010 to 0.019	-0.006^**^	0.002	-0.011 to -0.002
A-sLifestyle-Dep	0.004	0.003	0.000 to 0.014	-0.020	0.012	-0.048 to 0.000
B-iLifestyle-Dep	0.020^***^	0.003	0.015 to 0.026	-0.010^**^	0.003	-0.016 to -0.004
B-sLifestyle-Dep	0.003	0.003	-0.001 to 0.011	-0.014	0.012	-0.039 to 0.005
C-iLifestyle-Dep	0.010^***^	0.002	0.006 to 0.015	-0.004^*^	0.002	-0.008 to -0.002
C-sLifestyle-Dep	-0.001	0.003	-0.008 to 0.004	0.004	0.011	-0.017 to 0.027

ACEs, Adverse Childhood Experiences. A, Conventional adverse childhood experiences; B, Expanded adverse childhood experiences; C, New adverse childhood experiences. Dep, Depression. iLifestyle, Lifestyle intercept. sLifestyle, Lifestyle slope. ^***^ *P*<.001, ^**^ *P*<.01, ^*^ *P*<.05.

The indirect association of conventional ACEs with the depression intercept through baseline lifestyle was 0.014 (*P* <.001), accounting for 7.8% of the total effect (= 0.014/0.178), and the indirect association with the depression slope was -0.006 *(P* <.01), accounting for 21.4% of the total effect (=-0.006/-0.028). The direct effect of conventional ACEs on the depression intercept was 0.159 (*P* <.001). Similarly, For expanded ACEs, baseline lifestyle mediated 8.6% of the total effect on depression intercept (= 0.020/0.230). New ACEs showed smaller but significant mediation proportions, with lifestyle explaining 7.3% of the intercept effect (=0.010/0.136). Notably, expanded ACEs demonstrated the strongest mediation through lifestyle factors, with proportion mediated exceeding conventional ACEs by 0.8 percentage points (8.6% vs. 7.8%) and new ACEs by 1.3 percentage points (8.6% vs. 7.3%) in depression intercept effects.

## 4 Discussion

Although an increasing number of studies have explored the link between ACEs and depression, most of the findings come from Western countries, and relatively few studies have examined the longitudinal relationship between ACEs and depression in middle-aged and elderly individuals in non-Western countries, particularly focusing on the mediating role of lifestyle factors. To our knowledge, this is the first study using longitudinal data from a large national representative cohort to explore the mediating effect of lifestyle in the relationship between ACEs and depression. Our findings reveal several important insights into the complex relationship between early-life adversity, lifestyle, and depression trajectories over time.

First, the observed age-related increase in depressive symptoms coupled with improvements in lifestyle factors presents an intriguing paradox. While the worsening of depressive symptoms aligns with previous research on life course theory (44), the concurrent improvement in lifestyle factors may reflect increased health awareness or access to healthcare services among older adults in China (45, 46). However, these trends should be interpreted cautiously in light of potential methodological influences. First, cohort effects may partially explain the observed patterns: younger participants entering older age groups during the study period (2013-2020) may have been exposed to different socioeconomic conditions (e.g., rapid urbanization, healthcare reforms) compared to older cohorts, potentially confounding age-related trajectories. Second, survivor bias could play a role, as individuals with severe depression or unhealthy lifestyles may have been more likely to drop out of the study or die before subsequent waves, leading to an overestimation of lifestyle improvements and underestimation of depressive symptom severity in later waves. This finding suggests that merely improving lifestyle may be insufficient to counteract the age-related increase in depressive symptoms, highlighting the need for more comprehensive mental health interventions in this population.

Second, we found that individuals with more ACEs had higher baseline levels of depressive symptoms, which is consistent with the extensive literature documenting the long-term mental health consequences of childhood adversity (5, 18). The quality of parental care, cognitive stimulation, and socioeconomic status during early child development have been shown to affect brain morphology and functionality throughout the life course (47). Some studies on the neurophysiological mechanisms between childhood adversity and psychopathology suggest that ACEs may increase the neurobiological sensitivity to stress, thereby increasing the risk of developing depression (48–50). However, ACEs were not associated with the rate of change in depressive symptoms over time, suggesting that while early-life adversity may establish a higher baseline risk for depression, it does not necessarily accelerate the progression of depressive symptoms in later life. This pattern may reflect psychological adaptation mechanisms in later adulthood. For example, older adults with ACEs may develop compensatory coping strategies (e.g., emotion regulation, social support-seeking) over time to mitigate the ongoing impact of early trauma on mental health decline (51). This finding partially supports our research hypothesis 1.

Finally, through mediation analysis, we found that ACEs were associated with poorer baseline lifestyle levels, which, in turn, were linked to higher baseline depressive symptoms. This finding emphasizes that lifestyle is a modifiable pathway through which early life adversity may affect later mental health outcomes. Furthermore, the indirect effect of ACEs on the slope of depressive symptoms through lifestyle factors suggests that improvement in lifestyle may help mitigate the progression of depressive symptoms over time. This underscores the potential value of lifestyle interventions, particularly for individuals with a history of childhood adversity.

Notably, in terms of the ACEs dimension, the expanded ACEs (unsafe living environment, peer bullying, and poor childhood friendships) exhibited the strongest mediating effect. This finding suggests that adversities related to social and environmental factors during childhood may have a particularly strong influence on lifestyle behaviors and, consequently, mental health outcomes in later life. This is consistent with Samaah’s findings on the childhood social living environment and activity behaviors of children across 12 countries worldwide (52). In contrast, conventional ACEs and new ACEs showed smaller but still significant mediating effects. This differences highlight the importance of considering the specific nature of childhood adversities when examining their long-term consequences and designing targeted interventions.

### 4.1 Implication for practice and policy

Our findings have important implications for clinical practice and public health interventions. In China, individuals with a higher incidence of ACEs among middle-aged and elderly people exhibit more late-onset depressive symptoms. This underscores the importance of addressing ACEs at the societal and familial levels to promote long-term mental health and well-being. Policymakers and communities should prioritize initiatives to reduce exposure to adverse experiences, such as strengthening social support systems, promoting safe environments, and raising public awareness of the long-term consequences of early life adversity. To operationalize these goals, we recommend integrating ACE screening tools (e.g., WHO ACE-IQ adapted for older adults) into routine health assessments for middle-aged and elderly populations within primary care systems (53). Early identification of high-risk individuals could trigger targeted lifestyle interventions, such as community-based programs offering structured physical activity sessions, sleep hygiene workshops, and social engagement hubs tailored to rural and low-literacy populations. Additionally, we found that lifestyle factors partially mediated the relationship between ACEs and the initial severity and progression of depressive symptoms. This suggests that promoting healthy lifestyles could be an effective strategy to mitigate the long-term impact of childhood adversity on mental health, particularly for middle-aged and elderly individuals. Interventions targeting lifestyle factors, such as physical activity, diet, sleep, social connections, mindfulness-based stress reduction (MBSR) and social prescription initiatives, may be especially beneficial for individuals with a history of childhood adversity (54, 55). Among the proposed interventions, structured physical activity programs (e.g., Tai Chi) and sleep hygiene workshops may be the most feasible, as they align with the cultural context and functional capacities of rural older adults in China, and can be readily integrated into existing community health infrastructures.

### 4.2 Limitations

To the best of our knowledge, this is the first study to explore the mediating effect of lifestyle factors in the relationship between ACEs and depression in a nationally representative Chinese population. However, several potential limitations of this study should be acknowledged. First, the reliance on self-reported data may introduce recall bias, particularly for retrospective reports of childhood adversity, so caution is needed in interpreting the relationship between ACEs and depression. Second, While the CHARLS dataset is nationally representative of middle-aged and elderly Chinese individuals, the findings may not be fully generalizable to other populations. Examining the effects of different populations would help researchers to propose more targeted guidance measures, and this should be considered in future studies. Third, although our lifestyle score integrated multiple health-related behaviors, the dichotomization of continuous variables (e.g., defining physical activity as a binary threshold of >10 minutes per week, or sleep duration as >6 hours per night) may have reduced variability in the measurement. This approach, while simplifying interpretation and aligning with prior CHARLS-based studies (41), could attenuate statistical power to detect nuanced associations between lifestyle gradients and depression trajectories. Future studies using continuous or multi-category operationalization of these constructs may provide more granular insights. Fourth, while our longitudinal design allows for temporal ordering of variables, the observational nature of the data limits causal inference. Reverse causality remains a possibility; depressive symptoms may reciprocally influence lifestyle behaviors (e.g., reduced physical activity due to low motivation) rather than solely reflecting ACE-driven pathways. Future research could employ cross-lagged panel models or intervention studies to better disentangle the directionality of these associations. Finally, While we controlled for a range of demographic factors, such as age, gender, marital status, and education level, there may still be other confounding variables that were not accounted for in the analysis. These could include genetic factors, environmental influences, or other psychosocial variables that may contribute to depression and lifestyle behaviors, affecting the strength and direction of the observed relationships. Future studies should consider a wider range of potential factors.

### 4.3 Conclusion

In conclusion, this study provides valuable insights into the complex relationship between ACEs, lifestyle factors, and depression in middle-aged and elderly individuals. The results suggest that while ACEs contribute to higher baseline depression severity, lifestyle factors play a critical mediating role in both the initial severity and progression of depressive symptoms. The findings highlight the importance of integrating lifestyle interventions into mental health strategies for individuals with a history of childhood adversity, particularly those exposed to expanded ACEs. We call on policymakers and mental health professionals to implement immediate actions: integrate ACE screening into primary care for older adults, and scale up community-based programs tailored to rural populations—steps aligned with China’s Healthy Aging goals to disrupt the ACEs-depression cycle. Future research should explore additional pathways through which ACEs influence mental health outcomes and further evaluate the effectiveness of lifestyle interventions in mitigating these effects.

## Data Availability

The datasets presented in this study can be found in online repositories. The names of the repository/repositories and accession number(s) can be found below: https://charls.charlsdata.com/index/zh-cn.html.

## References

[1] Xu J Han G Xu X. Adverse childhood experiences and 10-year depressive-symptoms trajectories among middle-aged and older adults in China: A population-based cohort study. Front Public Health. (2024) 12:1455750. doi: 10.3389/fpubh.2024.1455750, PMID: 39717034 PMC11663716

[2] Fang EF Scheibye-Knudsen M Jahn HJ Li J Ling L Guo H. A research agenda for aging in China in the 21st century. Ageing Res Rev. (2015) 24:197–205. doi: 10.1016/j.arr.2015.08.003, PMID: 26304837 PMC5179143

[3] Qiu Q-W Qian S Li J-Y Jia R-X Wang Y-Q Xu Y. Risk factors for depressive symptoms among older chinese adults: A meta-analysis. J Affect Disord. (2020) 277:341–6. doi: 10.1016/j.jad.2020.08.036, PMID: 32861154

[4] Blazer DG. Depression in late life: review and commentary. J Gerontology: Med Sci. (2003) 58A:249–65. doi: 10.1093/gerona/58.3.M249, PMID: 12634292

[5] Yin H Zhu Y Tan L Zhong X Yang Q. The impact of adverse childhood experiences on depression in middle and late life: A national longitudinal study. J Affect Disord. (2024) 351:331–40. doi: 10.1016/j.jad.2024.01.132, PMID: 38244797

[6] Vos T Flaxman AD Naghavi M Lozano R Michaud C Ezzati M. Years lived with disability (Ylds) for 1160 sequelae of 289 diseases and injuries 1990–2010: A systematic analysis for the global burden of disease study 2010. Lancet. (2012) 380:2163–96. doi: 10.1016/s0140-6736(12)61729-2, PMID: 23245607 PMC6350784

[7] Chapman DP Perry GS. Depression as a major component of public health for older adult. Prev Chronic Dis. (2008) 5:1–9., PMID: 18082011 PMC2248771

[8] Naghavi M. Global, Regional, and National Burden of Suicide Mortality 1990 to 2016: Systematic Analysis for the Global Burden of Disease Study 2016. Bmj. (2019) 364:194–204. doi: 10.1136/bmj.l94, PMID: 31339847 PMC6598639

[9] Doraiswamy PM Khan ZM Donahue RMJ Richard NE. The spectrum of quality-of-life impairments in recurrent geriatric depression. J Gerontology: Med Sci. (2002) 57A:M134–M7. doi: 10.1093/gerona/57.2.M134, PMID: 11818434

[10] Tudoran M Tudoran C Pop GN Bredicean C Giurgi-oncu C. The contribution of individual mental health and socioeconomic status to the evolution of elderly patients with chronic heart failure. Riv Psichiatr. (2021) 56:107–12. doi: 10.1708/3594.35769, PMID: 33899832

[11] Lu S Liu T Wong GHY Leung DKY Sze LCY Kwok W-W. Health and social care service utilisation and associated expenditure among community-dwelling older adults with depressive symptoms. Epidemiol Psychiatr Sci. (2021) 30:1–10. doi: 10.1017/s2045796020001122, PMID: 33526166 PMC8057460

[12] Sachs-Ericsson NJ Rushing NC Stanley IH Sheffler J. In my end is my beginning: developmental trajectories of adverse childhood experiences to late-life suicide. Aging Ment Health. (2015) 20:139–65. doi: 10.1080/13607863.2015.1063107, PMID: 26264208

[13] Wang Q Wu H-T Liu C Huang X-T Xu X-R Wu B-Y. A pathway analysis of the impact of childhood domestic violence on depression in middle-aged and elderly people from the perspective of life course. Child Abuse Negl. (2023) 145:1–9. doi: 10.1016/j.chiabu.2023.106403, PMID: 37633219

[14] Felitti VJ Anda RF Nordenberg D Williamson DF Spitz AM Edwards V. Relationship of childhood abuse and household dysfunction to many of the leading causes of death in adults: the adverse childhood experiences (Ace) study. Am J Prev Med. (1998) 14:245–58. doi: 10.1016/S0749-3797(98)00017-8, PMID: 9635069

[15] Gelaye B Rondon MB Araya R Williams MA. Epidemiology of maternal depression, risk factors, and child outcomes in low-income and middle-income countries. Lancet Psychiatry. (2016) 3:973–82. doi: 10.1016/s2215-0366(16)30284-x, PMID: 27650773 PMC5155709

[16] McLaughlin KA Sheridan MA Humphreys KL Belsky J Ellis BJ. The value of dimensional models of early experience: thinking clearly about concepts and categories. Perspect psychol Sci. (2021) 16:1463–72. doi: 10.1177/1745691621992346, PMID: 34491864 PMC8563369

[17] Cheong EV Sinnott C Dahly D Kearney PM. Adverse childhood experiences (Aces) and later-life depression: perceived social support as a potential protective factor. BMJ Open. (2017) 7:1–11. doi: 10.1136/bmjopen-2016-013228, PMID: 28864684 PMC5588961

[18] Yin H Qiu X Zhu Y Yang Q. Adverse childhood experiences affect the health of middle-aged and older people in China: the multiple mediating roles of sleep duration and life satisfaction. Front Psychiatry. (2023) 14:1092971. doi: 10.3389/fpsyt.2023.1092971, PMID: 37032944 PMC10073436

[19] Fuller-Thomson E B Katz R Phan V T Liddycoat J PM Brennenstuhl S. The long arm of parental addictions: the association with adult children’s depression in a population-based study. Psychiatry Res. (2013) 210:95–101. doi: 10.1016/j.psychres.2013.02.024, PMID: 23642525

[20] Hammen C Henry R Daley SE. Depression and sensitization to stressors among young women as a function of childhood adversity. J Consulting Clin Psychol. (2000) 68:782–7. doi: 10.1037/0022-006x.68.5.782, PMID: 11068964

[21] Case A Fertig A Paxson C. The lasting impact of childhood health and circumstance. J Health Economics. (2005) 24:365–89. doi: 10.1016/j.jhealeco.2004.09.008, PMID: 15721050

[22] Lei X Sun X Strauss J Zhang P Zhao Y. Depressive symptoms and ses among the mid-aged and elderly in China: evidence from the China health and retirement longitudinal study national baseline. Soc Sci Med. (2014) 120:224–32. doi: 10.1016/j.socscimed.2014.09.028, PMID: 25261616 PMC4337774

[23] Ford ES Anda RF Edwards VJ Perry GS Zhao G Li C. Adverse childhood experiences and smoking status in five states. Prev Med. (2011) 53:188–93. doi: 10.1016/j.ypmed.2011.06.015, PMID: 21726575

[24] Brugiavini A Buia RE Kovacic M Orso CE. Adverse childhood experiences and unhealthy lifestyles later in life: evidence from share countries. Rev Economics Household. (2022) 21:1–18. doi: 10.1007/s11150-022-09612-y

[25] Lopresti AL Hood SD Drummond PD. A review of lifestyle factors that contribute to important pathways associated with major depression: diet, sleep and exercise. J Affect Disord. (2013) 148:12–27. doi: 10.1016/j.jad.2013.01.014, PMID: 23415826

[26] Nurius PS Green S Logan-Greene P Longhi D Song C. Stress pathways to health inequalities: embedding aces within social and behavioral contexts. Int Public Health J. (2016) 8:241–56., PMID: 27274786 PMC4891624

[27] Chu J Raney JH Ganson KT Wu K Rupanagunta A Testa A. Adverse childhood experiences and binge-eating disorder in early adolescents. J Eating Disord. (2022) 10:168–74. doi: 10.1186/s40337-022-00682-y, PMID: 36384578 PMC9670461

[28] Sheffler JL Meng Z Sachs-Ericsson N Caimary VG Patel J Pickett S. Sleep Quality as a Critical Pathway between Adverse Childhood Experiences and Multimorbidity and the Impact of Lifestyle. J Aging Health. (2024) 37:167–81. doi: 10.1177/08982643241237832.10.1177/08982643241237832, PMID: 38447525

[29] MamplekouEBountzioukaVPsaltopoulouTZeimbekisATsakoundakisNPapaerakleousN. Urban environment, physical inactivity and unhealthy dietary habits correlate to depression among elderly living in eastern mediterranean islands: the medis(Medierranean islands elderly)Study. J Nutrition Health Aging. (2010) 14:449–55. doi: 10.1007/s12603-010-0091-0, PMID: 20617287

[30] Sánchez-Villegas A Toledo E de Irala J Ruiz-Canela M Pla-Vidal J Martínez-González MA. Fast-food and commercial baked goods consumption and the risk of depression. Public Health Nutr. (2011) 15:424–32. doi: 10.1017/s1368980011001856, PMID: 21835082

[31] Anda RF Croft JB Felitti VJ Nordenberg D Giles WH Williamson DF. Adverse childhood experiences and smoking during adolescence and adulthood. JAMA. (1999) 282:1652–8. doi: 10.1001/jama.282.17.1652, PMID: 10553792

[32] Azevedo Da Silva M Singh-Manoux A Brunner EJ Kaffashian S Shipley MJ Kivimäki M. Bidirectional association between physical activity and symptoms of anxiety and depression: the whitehall ii study. Eur J Epidemiol. (2012) 27:537–46. doi: 10.1007/s10654-012-9692-8, PMID: 22623145 PMC4180054

[33] Baron RM Kenny DA. The moderator-mediator variable distinction in social psychological research: conceptual, strategic, and statistical considerations. J Pers Soc Psychol. (1986) 51:1173–82. doi: 10.1037/0022-3514.51.6.1173 3806354

[34] Grant KE Compas BE Thurm AE McMahon SD Gipson PY Campbell AJ. Stressors and child and adolescent psychopathology: evidence of moderating and mediating effects. Clin Psychol Rev. (2006) 26:257–83. doi: 10.1016/j.cpr.2005.06.011, PMID: 16364522

[35] Raison CL Capuron L Miller AH. Cytokines sing the blues: inflammation and the pathogenesis of depression. Trends Immunol. (2006) 27:24–31. doi: 10.1016/j.it.2005.11.006, PMID: 16316783 PMC3392963

[36] Sarris J O’Neil A Coulson CE Schweitzer I Berk M. Lifestyle medicine for depression. BMC Psychiatry. (2014) 14:1–27. doi: 10.1186/1471-244X-14-107, PMID: 24721040 PMC3998225

[37] Green JG McLaughlin KA Berglund PA Gruber MJ Sampson NA Zaslavsky AM. Childhood adversities and adult psychiatric disorders in the national comorbidity survey replication I: associations with first onset of dsm-iv disorders. Arch Gen Psychiatry. (2010) 67:113–23. doi: 10.1001/archgenpsychiatry.2009.186, PMID: 20124111 PMC2822662

[38] Monroe SM Harkness KL. Major depression and its recurrences: life course matters. Annu Rev Clin Psychol. (2022) 18:329–57. doi: 10.1146/annurev-clinpsy-072220-021440, PMID: 35216520

[39] Zhao Y Hu Y Smith JP Strauss J Yang G. Cohort profile: the China health and retirement longitudinal study (Charls). Int J Epidemiol. (2012) 43:61–8. doi: 10.1093/ije/dys203, PMID: 23243115 PMC3937970

[40] Boey KW. Cross-validation of a short form of the ces-D in chinese elderly. Int J Geriatric Psychiatry. (1999) 14:608–17. doi: 10.1002/(SICI)1099-1166(199908)14:8<608::AID-GPS991>3.0.CO;2-Z, PMID: 10489651

[41] Liu Y Cui J Cao L Stubbendorff A Zhang S. Association of depression with incident sarcopenia and modified effect from healthy lifestyle: the first longitudinal evidence from the charls. J Affect Disord. (2024) 344:373–9. doi: 10.1016/j.jad.2023.10.012, PMID: 37805156

[42] Rojo-Wissar DM Sosnowski DW Ingram MM Jackson CL Maher BS Alfano CA. Associations of adverse childhood experiences with adolescent total sleep time, social jetlag, and insomnia symptoms. Sleep Med. (2021) 88:104–15. doi: 10.1016/j.sleep.2021.10.019, PMID: 34742038 PMC9012105

[43] Zheng X Cui Y Xue Y Shi L Guo Y Dong F. Adverse childhood experiences in depression and the mediating role of multimorbidity in mid-late life: A nationwide longitudinal study. J Affect Disord. (2022) 301:217–24. doi: 10.1016/j.jad.2022.01.040, PMID: 35031336

[44] Glen H Elder J. The life course as development theory. Child Dev. (1998) 69:1–12., PMID: 9499552

[45] Kim K Shin S Kim S Lee E. The relation between ehealth literacy and health-related behaviors: systematic review and meta-analysis. J Med Internet Res. (2023) 25:1–15. doi: 10.2196/40778, PMID: 36716080 PMC9926349

[46] Baskerville NB Azagba S Norman C McKeown K Brown KS. Effect of a digital social media campaign on young adult smoking cessation. Nicotine Tobacco Res. (2016) 18:351–60. doi: 10.1093/ntr/ntv119, PMID: 26045252

[47] Antoniou G Lambourg E Steele JD Colvin LA. The effect of adverse childhood experiences on chronic pain and major depression in adulthood: A systematic review and meta-analysis. Br J Anaesthesia. (2023) 130:729–46. doi: 10.1016/j.bja.2023.03.008, PMID: 37087334 PMC10251130

[48] Herzog JI Schmahl C. Adverse childhood experiences and the consequences on neurobiological, psychosocial, and somatic conditions across the lifespan. Front Psychiatry. (2018) 9:420. doi: 10.3389/fpsyt.2018.00420, PMID: 30233435 PMC6131660

[49] McIntyre RS Soczynska JK Liauw SS Woldeyohannes HO Brietzke E Nathanson J. The association between childhood adversity and components of metabolic syndrome in adults with mood disorders: results from the international mood disorders collaborative project. Int J Psychiatry Med. (2012) 43:165–77. doi: 10.2190/PM.43.2.e, PMID: 22849038

[50] Heim C Shugart M Craighead WE Nemeroff CB. Neurobiological and psychiatric consequences of child abuse and neglect. Dev Psychobiology. (2010) 52:671–90. doi: 10.1002/dev.20494, PMID: 20882586

[51] Yu JJ Zhang Z. Long-term impact of adverse childhood experiences and perceived social support on depression trajectories. J Affect Disord. (2025) 369:255–64. doi: 10.1016/j.jad.2024.09.170, PMID: 39341289

[52] Sullivan SM Broyles ST Barreira TV Chaput J-P Fogelholm M Hu G. Associations of neighborhood social environment attributes and physical activity among 9–11 year old children from 12 countries. Health Place. (2017) 46:183–91. doi: 10.1016/j.healthplace.2017.05.013, PMID: 28544991

[53] Organization WH. Adverse childhood experiences international questionnaire (Ace-iq) (2018). Available online at: http://www.who.int/violence_injury_prevention/violence/activities/adverse_childhood_experiences/en/ (Accessed April 4, 2024).

[54] Alsubaie M Abbott R Dunn B Dickens C Keil TF Henley W. Mechanisms of action in mindfulness-based cognitive therapy (Mbct) and mindfulness-based stress reduction (Mbsr) in people with physical and/or psychological conditions: A systematic review. Clin Psychol Rev. (2017) 55:74–91. doi: 10.1016/j.cpr.2017.04.008, PMID: 28501707

[55] Drinkwater C Wildman J Moffatt S. Social prescribing. BMJ. (2019) 364:1–5. doi: 10.1136/bmj.l1285, PMID: 30923039

