# Supplementary material for: The longitudinal relationships among adverse childhood experiences, lifestyle, and late-life depression: a latent growth mediation model
Source: Front Psychiatry. 2025 Jul 29;16:1581908. doi: 10.3389/fpsyt.2025.1581908 (PMC12339450; doi:10.3389/fpsyt.2025.1581908)
Supplement: Supplementary file 1 [file SupplementaryFile1.docx]

Supplementary Material

**Table 1** Questionnaire Items and Percentage of Each ACE

| Dimension | Type of ACEs | Questions | Percentage |
| --- | --- | --- | --- |
| Childhood abuse and neglect | Physical abuse | When you were growing up, did your female/male guardian ever hit you? Was that often^a^, sometimes^a^, rarely, or never? | 28.88% |
|  | Emotional neglect | How much love and affection did your female guardian give you while you were growing up? Often, sometimes, rarely^a^, or never^a^? | 26.28% |
|  |  | How much effort did your female guardian put into watching over you? A lot, some, a little^a^, or not at all^a^? |  |
|  | Domestic violence | Have your father/mother ever beat up your mother/father? Often^a^, sometimes^a^, rarely, or never? | 8.68% |
|  | Family crime | During the years you were growing up, have your female/male guardian involved in criminal activities like burglary or selling stolen property? Yes^a^ or no? | 0.36% |
|  |  | During the years you were growing up, have your female/male guardian ever been arrested or sent to prison? Yes^a^ or no? |  |
|  | Mental illness in the family | During the years you were growing up, had your female/male guardian showed continued signs of sadness or depression? All time^a^, most^a^, some, or a little time of childhood? | 12.65% |
|  |  | Did your female/male guardian have abnormality of mind when you were young? Yes^a^ or no？ |  |
|  | Household Substance Abuse | During the years you were growing up, did your female/male guardian ever have alcoholism or drug? Yes^a^ or no? | 6.92% |
|  | Parents divorced or separated | Were your biological parents divorced(including long separation due to emotional problems)? Yes^a^ or no? | 0.74% |
| Expanded ACEs | Unsafe living conditions | Was it safe being out alone at night in the neighborhood where you lived as a child? Is it very safe, somewhat safe, not very safe^a^ or not safe at all^a^? | 8.13% |
|  | Peer bullying | When you were a child, how often were you picked on or bullied by kids in your neighborhood? Is it often^a^, sometimes^a^, rarely, or never? | 16.16% |
|  |  | When you were a child, how often were you picked on or bullied by kids in your school? Is it often^a^, sometimes^a^, rarely or never? |  |
|  | Bad friendship experiences | When you were a child, how often did you feel lonely for not having friends? Is it often^a^, sometimes^a^, not very often or never? | 30.13% |
|  |  | When you were a child, did you often have a group of friends that you felt comfortable spending time with? Is it often, sometimes, not very often^a^ or never^a^? |  |
|  |  | When you were a child, did you have a good friend? Yes or no^a^? |  |
| New ACEs | Parental death^b^ | Either of the parents was dead before participant was 17 years? Yes^a^ or no? | 16.91% |
|  | Death of a sibling^c^ | Any of the siblings was dead before participant was 17 years? Yes^a^ or no? | 16.57% |
|  | Parental disability | Did your female/male guardian have a long time be sick on bed when you were young? Yes^a^ or no? | 21.20% |
|  |  | Did your female/male guardian have a serious deformity when you were young? Yes^a^ or no? |  |

ACEs=adverse childhood experiences

^a^The answer indicates the ACE threshold.

^b^Calculated based on dates of birth and their parental death.

^c^Calculated based on dates of birth and their sibling’s death.

**Fig.1** Prevalence of ACEs in the sample (total ACEs scores and dimensions). Note: X-axis, scores of ACEs, and dimensions. Y-axis, number of samples.

**Table 2** Unstandardized parameter estimates for unconditional LGCM for depression and lifestyle

| **Parameter** | **depression** | |  | **Lifestyle** | |
| --- | --- | --- | --- | --- | --- |
|  | Estimate | SE |  | Estimate | SE |
| Mean |  |  |  |  |  |
| Intercept | 17.837^***^ | 0.053 |  | 3.216^***^ | 0.013 |
| Slop | 0.192^***^ | 0.008 |  | 0.081^***^ | 0.002 |
| Variance |  |  |  |  |  |
| Intercept | 20.675^***^ | 0.453 |  | 1.142^***^ | 0.028 |
| Slope | 0.170^***^ | 0.013 |  | 0.006^***^ | 0.001 |
| Covariance |  |  |  |  |  |
| Intercept↔Slope | -0.177^**^ | 0.073 |  | -0.014^***^ | 0.003 |

^**^*P*<0.01, ^***^*P*<0.001
